# Heartache in the Heartland: Unraveling the Social Roots of Deaths of Despair in Kentucky

**DOI:** 10.13023/jah.0701.03

**Published:** 2025-05-01

**Authors:** Lyndsey K. Blair, Madeline M. Tomlinson, Michele Abee-Biskis, Jessica Hume, Gabri Warren

**Affiliations:** Bellarmine University; Bellarmine University; Bellarmine University; Bellarmine University; Bellarmine University

**Keywords:** Appalachia, Addiction, Deaths of Despair, Socioeconomic Status

## Abstract

**Introduction:**

Deaths of despair (DoD), encompassing suicides, drug overdoses, and alcohol-related liver diseases, have emerged as a critical public health crisis in the United States, with their rise particularly pronounced from 1995 to 2013 and exacerbated by the COVID-19 pandemic. Kentucky, grappling with high rates of substance use disorder, poor mental health, and economic hardship, is at the forefront of this issue, particularly in its rural and Appalachian regions.

**Purpose:**

This study explores the social determinants contributing to DoD in Kentucky, focusing on economic and social factors that influence rising rates of suicide, drug overdose, and alcohol-related liver disease. The goal is to provide evidence to guide policy and intervention strategies.

**Methods:**

An ecological study was conducted across 120 Kentucky counties from 2011 – 2020. DoD mortality data were sourced from the CDC WONDER database, and socioeconomic variables from the American Community Survey. Principal Component Analysis (PCA) reduced 10 county-level socioeconomic variables. Poisson regression estimated associations between socioeconomic principal component scores and DoD mortality, adjusting for confounders like age, and racial demographics.

**Results:**

The median DoD mortality rate was 59.7 per 100,000 people, with geographic variation. Three principal components explained 78.4% of the variance in socioeconomic factors. Counties with extreme socioeconomic disadvantages (low education, high poverty, high disability, high unemployment) were strongly associated with higher DoD rates (RR=1.07; 95% CI=1.02–1.12).

**Implications:**

Extreme socioeconomic disadvantage is a key predictor of DoD rates in Kentucky. These findings can inform public health interventions and policy changes targeting high-risk areas, especially rural and Appalachian regions.

## INTRODUCTION

Phenomena christened “deaths of despair” (DoD) are deaths caused by suicide, drug overdose, and alcohol related liver disease, and have been identified as a public health crisis.^1^ The inception of this term followed a substantial increase of DoD across the United States (U.S.) from 1995 to 2013. This increase has continued to rise with added pressures from the COVID-19 pandemic.^2^ However, the high burden of DoD is not fully explained by the pandemic alone. Recent increases in these types of death draw awareness to consistent drops in the U.S. average life expectancy, a change beginning in 2015.^3^–^4^ The social determinants of health (SDOH) – the conditions in which people live, work, and play – hold a critical role in influencing the burden of DoD, highlighting the complex interplay between health outcomes and social conditions.

Kentucky is a critical focus for studying DoD because the state consistently ranks among the highest in the nation for rates of substance use disorder, poor mental health outcomes, and socioeconomic challenges.^5^ These factors place residents at an elevated risk of DoD, yet the specific social conditions underlying this crisis remain under-explored. The state's economic decline, particularly in rural and Appalachian regions, mirrors conditions observed in other areas which have experienced high rates of DoD, such as Appalachian Pennsylvania.^6^ By examining Kentucky, researchers and clinicians can better understand the ways that local social determinants like income inequality, lack of job availability, high dependability on federal aid, high dependability on state aid, limited access to healthcare, rural poverty rates, low high school graduation rates, and food insecurity all contribute to the rising rates of DoD. In summary, Kentucky provides a case study for understanding how economic, social, cultural, and healthcare factors contribute to DoD. Studying this state could offer important insights that are relevant not just for Kentucky, but for other rural or economically distressed regions facing similar challenges.

The objective of this study is to explore the underlying social determinants contributing to DoD in Kentucky, with a particular emphasis on the roles of economic and social factors that have been historically understudied. Understanding the local context is crucial for identifying actionable interventions to reduce the burden of DoD in this state. By shedding light on the unique social and economic challenges faced by Kentucky residents, this study aims to provide evidence that could inform local and federal policy interventions, ultimately contributing to efforts to reverse the negative trends in U.S. life expectancy.

## METHODS

### Outcome: County DoD Mortality Rates

This ecological study of Kentucky examined the association of county-level socioeconomic status (SES) patterns with county-level DoD mortality rates between 2011 – 2020. An ecological study was chosen because it allows for group-level analysis to identify potential associations, which can then be explored further with more detailed studies. Researchers extracted annual DoD mortality rates in each county from 2011 – 2020 using the CDC Wide-ranging Online Data for Epidemiologic Research (CDC WONDER) database on multiple cause-of-death of Kentucky residents. DoD mortality was classified with the International Statistical Classification of Diseases, 10th revision underlying cause of death codes X50-X84, Y87.0, *U03 (suicide deaths); X40-X45, Y10-Y15 (accidental and unintentional overdose/poisoning deaths); K70, K73, K74 (chronic liver disease and cirrhosis deaths). The analysis included all counties in Kentucky (n=120).

### Exposure and Covariates from the American Community Survey

The American Community Survey (ACS) is an annual survey on demographic, social, economic, and housing factors conducted by the U.S. Census Bureau from a large sample of U.S. addresses. Researchers linked the county-level 5-year estimates for 2016 – 2020 ACS characteristics to the county-level DoD mortality rates for Kentucky. County-level demographic and social factors considered were age, race, ethnicity, marital status, educational attainment, grandparent responsibility for grandchildren, family structure, physical disability, and vacant housing. Economic factors evaluated included income inequality, poverty, health insurance coverage, food stamp eligibility, and employment.

### Statistical Analyses

Counties were divided into tertiles based on their mortality rates per 100,000. For easier interpretation of socioeconomic principal components (PCs), researchers selected a reduced set of 10 SES variables to include in the principal component analysis (PCA) based on data observations from Table 1 and a review of the literature to include SES variables most relevant to DoD.^8^–^14^ Bivariate intercorrelation patterns of 10 socioeconomic variables were computed using Pearson correlation coefficients, with many statistically correlated with one other **(**Table 2). Strong variable intercorrelations prompted the use of the PCA to identify a reduced set of socioeconomic patterns across 120 Kentucky counties. Poisson regression was used to estimate the associations between the socioeconomic PC scores with DoD mortality rates **(**Table 3).

### Principal Component Analysis (PCA)

**Figure 1a-1c:**
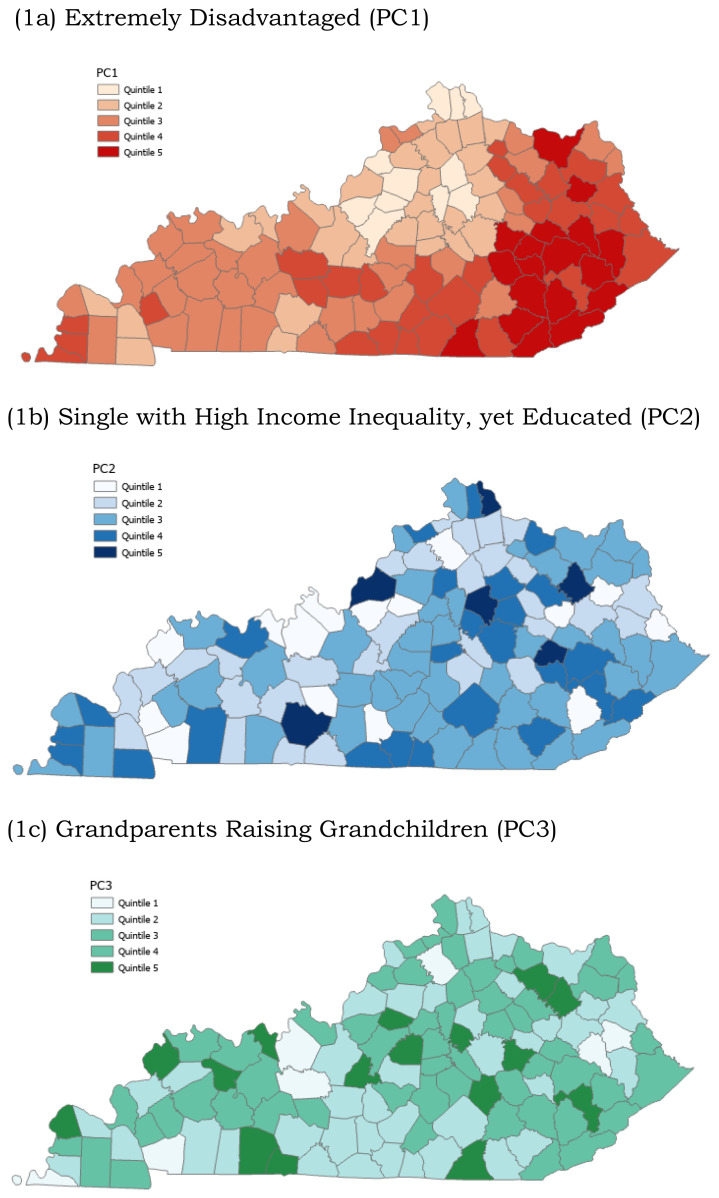
Kentucky Maps Showing the Spatial Distribution of Quintiles of Principal Component Scores

PCA is a data reduction technique frequently used in neighborhood-level research.^7^ Researchers chose PCA for this study to reduce the dimensionality of the 10 county-level socioeconomic variables and provide an empirical summary of the total variance explained by these variables. The PCA, conducted in SAS version 9.4, used PROC FACTOR to generate statistically uncorrelated PC scores that reflect patterns of social and economic variables. A scree plot, along with the criterion of retaining components that together explained at least 75% of the cumulative variance, was used to determine the number of principal components to include in subsequent outcome analyses **(**Fig. 1). PCA included county-level indicators such as the percentages of the population living below the poverty line, living with a disability, holding a bachelor’s degree or higher, receiving public health insurance, and possessing employment. Additionally percentages for grandparents responsible for grandchildren under 18, vacant housing units, married-couple families, people receiving food stamps, and the GINI index of income inequality were also included as a county-level indicators **(**Table 2). Factor loadings dictated that each PC should be labeled according to the variables with the largest absolute value of the coefficients **(**Table 2). PC scores mapped in quintiles using ArcGIS created data visualization showing the geospatial distribution of the socioeconomic PC scores across Kentucky, which health officials may find useful to find their counties’ score for the identified PC patterns **(**Fig. 1). The PC scores were standardized to z-scores before inclusion in the Poisson regression to allow for interpretation in terms of standard deviations.

### Outcome Analysis

Poisson regression applied to count data allowed estimation of relative risks (RRs) and 95% confidence intervals (CIs) for a 1-standard-deviation increase in each county-level PC z-score with a scaled deviance to account for overdispersion. The county’s DoD mortality count served as the dependent variable, and the natural log of county’s population size served as the offset term. Modifications to Poisson regression models included adjustments to account for confounding by population size; to account for residual confounding, researchers included a quadratic term for population size. Adjustment variables included county median age, percentage of white citizens, percentage of females, and percentage of non-Hispanic citizens. All statistical analyses were done in SAS version 9.4.

## RESULTS

In Kentucky, the median DoD mortality rate was 59.7 per 100,000 people (Interquartile Range [IQR]=50.4, 72.1). Compared to low DoD counties, counties with the highest DoD rates were slightly older with a larger white population, higher proportion of grandparents responsible for grandchildren, more disabled individuals, lower frequency of married households with families who tended to live below the poverty level, and were less likely to have a Bachelor’s degree or higher **(**Table 1).

### Principal Component Results

Three principal components explained 78.4% of the variation in ten county-level socioeconomic factors **(**Table 2) and were the location of the elbow in the scree plot **(**Fig. 1). High PC1 scores were correlated with lower education (Bachelor’s degree or higher, ρ=−0.717), high disability (ρ=0.805), lower employment (ρ= −0.933), higher public insurance (ρ=0.963), higher poverty (ρ=0.908), higher vacant housing (ρ=0.649), higher food stamps (ρ=.910), and higher income-inequality (ρ=0.481). A higher score on a PC indicates that the data point has a stronger association with the underlying features that contribute to that component. Counties with the highest PC1 scores have socioeconomic factors related to extreme disadvantages. Counties in the highest quintile of PC1 scores were distributed in the Appalachian Region of Kentucky **(**Fig. 1a).

High PC2 scores occurred in counties with a low proportion of married households with families (ρ= −0.682), high income inequality (ρ=0.644), yet higher education (Bachelor’s degree or higher, ρ=0.460). High PC2 scores indicate a profile of single individuals with high income inequality but have higher education than other poor counties. Counties in the highest quintile of PC2 were distributed throughout Kentucky, including the most urban counties, Jefferson and Fayette, and a cluster in the mid-eastern portion of Kentucky (Fig. 1b).

High PC3 scores were in counties with a higher proportion of grandparents responsible for grandchildren (ρ=0.856). High PC3 scores indicate a profile of grandparents responsible for grandchildren. Counties in the highest quintile of PC3 were distributed heterogeneously throughout Kentucky (Fig. 1c).

### Poisson Regression Results

Results are presented in Table 3. The extremely disadvantaged profile (PC1) was statistically associated with higher DoD rates in the unadjusted (RR=1.12 95% CI=1.06, 1.18) and adjusted models (RR=1.07; 95% CI=1.02,1.12). A high percentage of single individuals with high income inequality yet educated (PC2) was not associated with DoD rates in the unadjusted (RR=0.99; 95% CI=0.94, 1.05) nor adjusted models (RR=1.04; 95% CI=0.98, 1.10). Similarly, a high percentage of grandparents raising grandchildren (PC3) was not associated with DoD in unadjusted (RR=1.03; 95% CI=0.97, 1.08) nor adjusted models (RR=1.01; 95% CI=0.96, 1.05).

## DISCUSSION

The three most prominent socioeconomic profiles derived from the PCA were generally geographically located in the eastern portion of Kentucky, the Appalachian region of the state. Counties scoring high on the extremely disadvantaged county-level socioeconomic profile were statistically associated with DoD. County-level risk factors for DoD included less than a Bachelor’s degree education, high disability, low employment, high public health insurance, high poverty, high vacant housing, high prevalence of food stamps, and high income-inequality. Counties scoring high on the single adult or not married, high income inequality, yet educated profile, as well as counties scoring high in grandparents responsible for grandchildren, were not associated with DoD. This analysis demonstrated that multiple socioeconomic variables interact synergistically with DoD. This allowed for deeper insights into the combined effects of socioeconomic factors working together to impact DoD, rather than looking at individual factors independently of each other. Interventions should be considered in local contexts to address the variables outlined in each profile.

### Disability and DoD

Counties in PC1 had a significantly higher proportion of disabled citizens compared to the other PC scores. Studies have shown that adults with functional disabilities face elevated risks of DoD, including suicide, poisoning, and chronic liver disease.^15^,^16^ Additionally, individuals with intellectual disabilities (ID) are more vulnerable to the adverse effects of both prescribed and illicit substances, a crucial risk factor when evaluating substance use patterns.^17^,^18^

Prescription opioids remain essential for managing chronic and acute pain, but while most users follow medical guidelines, over 20% misuse these medications.^19^–^21^ Adults with disabilities are 1.5 times more likely to misuse opioids and are 40% less likely to receive treatment for opioid misuse compared to those without disabilities.^22^ This disparity may contribute to higher prescription opioid-related deaths in counties with a larger disabled population.

Research on alcohol consumption among individuals with ID has shown that overall alcohol use is lower than that of the general population. However, a significant proportion of those who do consume alcohol engage in misuse, increasing their risk of alcohol related death.^23^ Furthermore, persons with functional disabilities are at an increased risk for suicide-related outcomes compared to those without disabilities, such as suicidal ideation, suicide attempt, and death from suicide.^24^ The greater the number of physical and mental limitations, the higher the risk of suicide.^24^

When co-use of alcohol and opioids are present the risk of death from overdoses significantly increases.^22^ In addition, patients with chronic pain and mood disorders were both more likely than others to receive prescription opioids and were at a greater risk for suicide.^24^

### Socioeconomic Status and DoD

Due to the singular industry nature of the Eastern parts of the state, the decline in coal resulted in economic hardship and population decline in these areas.^25^ Lower SES factors, including low employment rates, high poverty levels, and high-income inequality, are often associated with high rates of DoD. Additionally, factors such as high rates of vacant housing and reliance on food stamps can exacerbate feelings of isolation and stress, as communities face disinvestment and reduced social cohesion. The combination of economic deprivation and lack of community support structures fosters environments where individuals are more prone to despair. Income inequality amplifies this effect, as those in lower SES brackets are more likely to feel the psychological burden of falling behind, which can lead to unhealthy coping mechanisms and increased DoD.^8^

PC1 had a high correlation with adults who have not achieved post-secondary degrees (e.g. Bachelor’s degree or higher). Lack of educational access, including the declining educational system and opportunities for vocational training, remains a significant predictor of DoD.^8^,^20^,^21^ Studies within the last decade have discovered that not only has opioid involvement in suicides doubled since 1999, but lack of educational attainment, alcohol and opioid misuse are linked to a heightened risk of suicidal ideation, attempts, and deaths.^22^,^24^

### Strengths and Limitations

The findings of this report are subject to several limitations. First, this was an ecological study using county-level data, which is susceptible to ecological fallacies, as the socioeconomic characteristics of individual overdose death cases are not available. Second, DoDs, particularly overdose deaths, may be underreported or misclassified, especially in rural or under-resourced areas. This could lead to an underestimation of actual mortality, potentially moving the study results closer to the null value. Misclassification is particularly likely when multiple substances are involved, complicating the interpretation of specific causes of death. Third, the cross-sectional nature of this study limits its ability to establish causality. While associations between socioeconomic factors and DoD were observed, it is difficult to determine whether these factors directly influenced mortality rates or if other variables were involved. Lastly, although the study considered various socioeconomic factors, deaths of despair are also influenced by cultural, family, and psychological factors, which are challenging to quantify at the community level. Factors such as community support, stigma, access to mental health services, and individual mental health history were not included, although they are important determinants of outcomes.

Despite these limitations, this study represents the first of its kind in Kentucky, a Commonwealth heavily impacted by DoDs and addiction. The large and diverse dataset provides a comprehensive representation of different socioeconomic groups, enhancing the generalizability of the findings. DoDs represent a significant public health challenge, and this study offers timely insights that can inform policy and intervention strategies. The potential impact of this research is significant, practical, and highly relevant.

## IMPLICATIONS

DoD, namely drug poisoning, suicide, and alcohol-related deaths have been a national public health concern since the turn of the century. Despite Kentucky being an epicenter of opioid misuse and DoD, there are limited studies assessing this public health issue. This study showed that socioeconomic county profiles were statistically associated with DoD rates in Kentucky. Identifying key risk factors, for DoD, such as mental health issues, economic hardship, or social isolation can help non-profits and policymakers create impactful interventions to assist vulnerable populations. Research can reveal gaps in mental health care and addiction treatment services, which is particularly relevant to Kentucky as the Commonwealth continues to experience challenges in accessing health care, especially in rural areas. This study can lead to increased investment in health resources, directly reducing DoD. Also, this study can guide policymakers in developing an evidence-based approach tailored to Kentucky’s unique challenges. For example, an initiative focusing on healthcare access, with an emphasis on mental health resources, and support for communities with high rates of substance disorders.

This study can also contribute to the public awareness of DoD. This work can be used to aid in the education of communities on the risk factors for DoD which will help Kentuckians to advocate for themselves and seek resources to help their communities. By establishing a baseline of understanding of DoD, Kentuckians will be able to better understand whether or not their interventions are contributing to a healthier community.

These risk-factor profiles may help federal agencies, state agencies, and local health departments tailor prevention strategies to curb the DoD epidemic based on their county's different socioeconomic profiles. Addressing these issues requires not only economic solutions but also robust public health interventions to provide evidence-based mental health and addiction support services to vulnerable populations. This study contributes to ongoing efforts to strengthen existing knowledge of the DoD epidemics and its contributing SES factors, which may promote the development of appropriate public health interventions targeted to high-risk populations.

**SUMMARY BOX**

**What is already known about this topic?**

Deaths of Despair are fatalities from drug overdoses, alcohol-related diseases, and suicide that have significantly impacted Kentucky. The crisis is driven by economic hardship, social isolation, and healthcare access issues, especially in Appalachian areas, and while progress is being made, it remains a pressing public health challenge.

**What is added by this report?**

Severe socioeconomic disadvantage is a strong predictor of Deaths of Despair in Kentucky. These insights can guide public health strategies and policy reforms focused on the most vulnerable areas, particularly in rural and Appalachian communities.

**What are the implications for future research?**

Future research enables focused interventions across Appalachia, such as improved access to telehealth support for those experiencing mental and physical challenges or substance use disorder.

## Figures and Tables

**Table 1:** County-Level Socioeconomic Characteristics by Groups of Deaths of Despair Mortality Rates per 100,000 People, Kentucky 2011–2020

	Tertile 1	Tertile 2	Tertile 3
**No. of Counties**	n=40	n=40	n=40
**Median death rate per 100,000 (IQR)**	45.5 (42.8, 50.4)	59.7 (56.4, 62.4)	80.5 (72.1, 84.2)
**% of married household families**	52.0 (6.4)	51.0 (4.5)	48.9 (5.0)
**% of grandparents responsible for grandchildren**	52.8 (14.8)	54.6 (12.1)	57.4 (13.8)
**% Bachelor’s degree or higher**	18.6 (7.6)	18.2 (7.7)	14.9 (6.8)
**% civilian noninstitutionalized population with a disability**	19.4 (4.9)	19.9 (4.3)	23.5 (6.4)
**% vacant housing units**	14.7 (6.4)	16.3 (7.2)	17.6 (5.9)
**% civilian noninstitutionalized population employed**	52.4 (7.0)	51.6 (7.7)	45.8 (9.8)
**% of all people receiving food stamps**	13.6 (5.2)	14.6 (5.3)	21.2 (8.7)
**% with public health insurance**	45.3 (8.2)	47.9 (8.0)	54.6 (11.4)
**% with income below poverty level**	17.5 (5.8)	18.3 (5.4)	23.5 (7.0)
**Gini Index of income inequality**	0.45 (0.04)	0.46 (0.04)	0.47 (0.04)
**County median age**	40.2 (3.6)	41.7 (2.7)	41.2 (2.5)
**% who are White**	90.7 (6.4)	92.3 (6.0)	94.9 (3.0)
**% who are female**	50.4 (1.7)	50.3 (1.8)	50.3 (1.8)
**% who are non-Hispanic**	96.9 (2.1)	97.5 (1.5)	98.2 (1.4)

NOTE:

Values are means (SD) unless otherwise noted.

**Table 2:** Principal Component (PC) Loadings Showing the Correlations Between the PC Scores and Socioeconomic Variables

	PC1	PC2	PC3
Eigenvalues	5.581	1.373	0.887
Proportion of variability explained	0.558	0.137	0.089
Cumulative proportion of variability explained	0.558	0.695	0.784
% Married with families	−0.416	**−0.682**	0.218
% Of grandparents responsible for grandchildren	0.378	0.257	**0.856**
% Bachelor’s degree or higher	**−0.717**	**0.460**	−0.102
% Civilian noninstitutionalized population with a disability	**0.805**	−0.222	0.030
% Vacant housing units	**0.649**	−0.322	−0.256
% Civilian non-institutionalized population employed	**−0.933**	0.169	0.036
% With public health insurance	**0.963**	−0.041	−0.029
% With income below poverty level	**0.908**	0.145	−0.049
Gini Index of Income Inequality	**0.481**	**0.644**	−0.160
% population receiving food stamps	**0.910**	0.102	0.022

NOTE:

Counties with high PC1 scores reflect socioeconomic factors related to extreme disadvantage; counties with high PC2 scores reflect a pattern of single, with high income inequality yet educated’ counties with PC3 scores reflect a pattern of grandparents raising grandchildren. Values in bold indicate component loadings >0.40.

**Table 3:** Adjusted Relative Risk (RR) Estimates (95% Confidence Intervals [CIs]) of Deaths of Despair in Kentucky Counties 2011–2020 (n=120)

	Model 1*, RR (95% CI)	Model 2†, RR (95% CI)	Model 3§, RR (95% CI)	Model 4¶, RR (95% CI)
**PC1**	1.12 (1.06, 1.18)	1.10 (1.04, 1.16)	1.07 (1.02, 1.12)	1.07 (1.02, 1.12)
**PC2**	0.99 (0.94, 1.05)	1.02 (0.96, 1.08)	1.04 (0.99, 1.09)	1.04 (0.98, 1.10)
**PC3**	1.03 (0.97, 1.08)	1.02 (0.97, 1.08)	1.01 (0.96, 1.05)	1.01 (0.96, 1.05)
**County median age**	--	1.08 (1.03, 1.14)	1.02 (0.97, 1.07)	1.02 (0.97, 1.07)
**% in county who are White**	--	--	1.12 (1.05, 1.19)	1.12 (1.05, 1.20)
**% in county who are non-Hispanic**	--	--	1.05 (0.99, 1.12)	1.05 (0.92, 1.12)
**% in county who are female**	--	--	--	1.00 (0.94, 1.06)

NOTE:

All estimates are for a 1-standard-deviation (SD) increase. SD for age is 3.01 years, SD for race is 5.59%, SD for non-Hispanic is 1.74%, and SD for female is 1.74%.

* Model 1 included the PCs, population size and a square term for population size.

† Model 2 included model 1 and county median age.

§ Model 3 included model 2 and percentage of the population that was White and percentage of the population that was non-Hispanic.

¶ Model 4 included model 3 covariates and additionally included the percentage of the population that was female.
